# Applying behavioural science to increase uptake of the NHS Health Check: a randomised controlled trial of gain- and loss-framed messaging in the national patient information leaflet

**DOI:** 10.1186/s12889-019-7754-5

**Published:** 2019-11-14

**Authors:** Natalie Gold, Caroline Durlik, Jet G. Sanders, Katherine Thompson, Tim Chadborn

**Affiliations:** 1 Public Health England, Wellington House, 133-155 Waterloo Rd, Lambeth, London, SE1 8UG UK; 2 Radcliffe Humanities, University of Oxford, Woodstock Road, Oxford, OX2 6GG UK; 3 Department of Psychological and Behavioural Science, London School of Economics and Political Science, Houghton St, Holborn, London, WC2A 2AE UK

**Keywords:** Framing, Invitations, Health behaviours, NHS Health Check, Patient information leaflets, Persuasive communication, Prevention behaviours

## Abstract

**Background:**

The NHS Health Check (NHSHC) is a national programme for the prevention of non-communicable diseases. Patients aged 40–74 without an existing cardiovascular-related condition should be invited quinquennially. Uptake is lower than anticipated. We assessed the impact on uptake of two new behaviourally-enhanced leaflets (with the current national leaflet as a control), enclosed with the invitation letter: the first trial on the leaflet.

**Methods:**

A double-blind three-armed randomized controlled trial was conducted. The new leaflets were shorter (two pages, instead of four); one was loss-framed (‘don’t miss out’) and the other was gain-framed (‘make the most of life’). The participants were patients from 39 practices in Lewisham and 17 practices in NE Lincolnshire, who were allocated to interventions using a random-number generator and received one of the leaflets with their invitation letter from April–September 2018. The outcome measure was uptake of an NHSHC by November 2018. The trial was powered to detect a 2% effect.

**Results:**

Uptake was 17.6% in the control condition (*n* = 3677), 17.4% in the loss-framed condition (*n* = 3664), and 18.2% in the gain-framed condition (*n* = 3697). Leaflet type was not a significant predictor of NHSHC uptake in a logistic regression that controlled for demographic variables, with GP practice as a random effect. Statistically significant predictors of uptake included location (higher uptake in Lewisham), age (increased age was associated with increased attendance) and sex (higher uptake in females). The Bayes Factor comparing the null to a hypothesis of differences between conditions was 416, which is extreme evidence in favour of the null hypothesis.

**Conclusion:**

There was no evidence for a meaningful effect of either a loss-framed or gain-framed behaviourally-informed leaflet type on uptake, which is surprising, given that behaviourally informed letters have improved uptake of NHSHCs. It is possible that people do not pay attention to leaflets that are enclosed with letters, or that the leaflet continues to support informed decision-making but this does not affect uptake.

**Trial registration:**

Clinicaltrials.gov, NCT03524131. Registered May 14, 2018. Retrospectively registered.

## Introduction

The National Health Service (NHS) Health Check is a national programme for the prevention of non-communicable diseases, such as stroke, kidney disease, heart disease, type 2 diabetes, and dementia [1]. All adults between 40 and 74 with no pre-existing vascular condition should receive an invitation to an NHS Health Check every 5 years. At the test, the following measures are recorded: age, gender, smoking status, family history of coronary heart disease, ethnicity, body mass index, cholesterol level, physical-activity level, alcohol use, 10 year risk of CVD using QRISK [2]. As a result, those patients with previously undiagnosed conditions can be put on a clinical pathway, and those who are at high risk of developing a condition can be offered lifestyle support and advice, in order to manage their risk. Furthermore, a key aspect of the programme is to encourage behaviour change for everyone in the general population who has sub-optimal diet, physical activity, tobacco or alcohol consumption. The programme has increased the detection of risk factors and disease, and research shows it has led to small reductions in the risk of cardiovascular disease in the general population [3]. Public Health England (PHE) and NHS England are taking action to support local areas to improve both uptake and effectiveness, in order to maximise the impact of the programme [4].

Before its roll-out in 2009, economic modelling showed that the NHS Health Check was likely to be effective and result in significant health improvements, based on an anticipated uptake of 75% [5]. This 75% figure was used in the economic modelling as an indicative figure because some screening programmes had set similar ambitions for take up. However, the national average uptake for 2014–2019 was 48% [6]. More recent microsimulation modelling shows that an increase in likelihood of attendance of 30% for each patient, compared to 2015 levels, would result in significant health gains [7]. The statutory duty to ensure that NHS Health Checks are offered to the eligible population lies with local authorities [1]. In 2013, PHE published an NHS Health Check Implementation Review and Action Plan; one of the action points was to ‘work with local authority NHS Health Check teams to test the impact of behavioural insight and marketing interventions on uptake’ [8], p.5.

There is flexibility in how local authorities commission the NHS Health Check programme, so a variety of invitation methods are used, but the most common is a letter from the patient’s GP accompanied by a patient information leaflet, supplied by Public Health England. Several trials have shown that applying behavioural science to the invitation letter can improve uptake [9–11]. The national invitation letter template has been updated based on their findings [12]. However, no studies until now have applied behavioural science to the NHS Health Check patient information leaflet.

Patient information leaflets are included alongside screening invitations to give patients information about the process and to present possible benefits and harms, so that patients can make an informed choice about whether to attend. PHE’s current NHS Health Check patient information leaflet is 4-sided, A5, and is intended to be sent with the invitation letter (a copy is included here as Additional file 1). Public facing information on the check is also published on the NHS Website [13]. The leaflet sets out the aims of the NHS Health Check and what a participant can expect at their appointment. It also explains the risk factors associated with cardiovascular disease.

The leaflet presents the benefits and harms of the NHS Health Check: it informs patients that the check has the potential to reduce their chances of developing certain conditions, but warns them that some people may be worried about the check and the impact of the results on their lifestyle. The leaflet can be ordered for free or downloaded online. Qualitative research commissioned during the development of the leaflet found that it was clearly written, visually appealing, and was seen as having just the right amount of information [14]. However, there was also feedback that the general messages about healthy lifestyles might be obscuring the call to attend the check, and in focus groups participants often did not read the back two pages of the leaflet [14].

There has been no previous research on the effect of NHS Health Check patient information leaflets on uptake. There has been research on patient information leaflets in screening programmes, but this has focussed on redesigning patient information leaflets in order to promote knowledge and informed choice [15–17]. A trial that sent an enhanced procedural instruction leaflet, telling people how to take a stool sample, found that more people returned their test kit for colorectal cancer screening [18]. This is the first trial that we know of to apply behavioural science to patient information leaflets with the aim of increasing attendance at an appointment, as well as the first trial on the NHS Health Check leaflet.

The literature on behavioural science and health behaviours suggests that the following insights from behavioural science could improve uptake of NHS Health Checks:

- *Simplification*: NHS Health Check invitation letters whose text was shortened and simplified, and which included clear behavioural instruction to book an NHS Health Check, succeeded in improving uptake [9, 10].
- *Social norms:* People are more likely to undertake a behaviour if they believe that others are also doing it [19]; using descriptive social norms has been successful in a number of settings, including tax compliance [20], reducing antibiotic prescribing [21], and increasing charitable giving [22].
- *Self-efficacy*: Beliefs about capability of performing specific behaviours in specific situations are predictive of successful health behaviour change [23] and setting small, achievable goals is a factor in the initiation and maintenance of health behaviour change [24, 25].

A revised leaflet might also be able to correct misunderstandings about the NHS Health Check: among qualitative studies of patient experience of the NHS Health Check process, there is a consensus that patients misunderstand the aim of the NHS Health Check and that this is a barrier to attendance [26–29]. A previous trial showed that uptake increased when some common myths that are barriers to uptake of the NHS Health Check were addressed in the invitation letter [11]. However, this insight is not a part of the current national invitation letter template. Barriers to attending NHS Health Checks (which could be addressed in the leaflet to improve uptake) include: patients having time constraints and competing priorities [30], not wanting to waste NHS resources if they are feeling well [26–28], being fatalist about health outcomes and thinking that learning their risks would not be helpful [26–28], and not wanting to be told off or receive unwelcome lifestyle advice [27].

Misunderstanding can also be a problem when patients attend, since some had the impression that they were going to get a broader and more comprehensive health check [27, 29], so their expectations were not met. Multiple studies have reported patients’ perception that more information at the point of invitation would be helpful [28, 29] and one reported that several interviewees suggested simplifying the information leaflet [26]. Two systematic reviews of patient experiences both concluded that there is a need for improved communication around the purpose of the NHS Health Check [30, 31]. These findings suggest that there is scope to optimise the information leaflet.

As well as using these insights to produce a behaviourally-enhanced leaflet, it may be possible to increase uptake by altering the framing of the leaflet, since people’s choices may depend on whether the outcomes of their decisions are framed as gains or losses. According to Prospect Theory, people are risk-seeking over losses but risk-averse over gains, and their decisions may change depending whether gains or losses are emphasized: framing in terms of losses leads to the choice of the relatively risky option, whereas framing in terms of gains leads to the choice of the relatively safe option [32]. In the context of health behaviours, it has been argued that Prospect Theory implies that when people perceive a health behaviour as involving a risk of a negative outcome (e.g., behaviours that detect the presence of a disease, such as attending screening appointments), loss-framed messages should be more effective; conversely when people perceive a health behaviour as involving a relatively low risk of a negative outcome (e.g., behaviours that prevent the onset of disease), then gain-framed messages should be more effective [33, 34].

Even though the aim of the NHS Health Check programme is preventive, it can be described as involving both detection (some of the tests involve biomarkers that could detect conditions such as type 2 diabetes or hypertension) as well as prevention. Consequently, it is not immediately clear whether gain-framed or a loss-framed messages should be more effective at increasing uptake. This study aimed to assess the impact on the uptake of NHS Health Checks of three different leaflets that were enclosed with the national template letter invitation: we tested two new behaviourally-enhanced leaflets, one gain-framed and one loss-framed, and compared them to each other and to the current national leaflet (which was our standard-practice control condition).

## Methods

### Study design

A double-blind three-armed randomized controlled trial was conducted. In order to find partners in local government for the trial, an advert was put in the operational supplement to the NHS Health Checks e-Bulletin, which goes out to subscribers. From the respondents, we chose to work with Lewisham and North East Lincolnshire. These two local authorities both have a centralised invitation procedure (although letters were signed from the patient’s GP, they were issued centrally, rather than by GP surgeries), which was important for ease of implementation of the trial. However, they are very different in terms of the locality and demographics, and in terms of the exact invitation procedure they use, which is important for the generalisability of results.

Patients in Lewisham and North East Lincolnshire were pseudo-randomised (see “Procedure and randomisation” for details) to receive either the current national leaflet, the new loss-framed leaflet, or the new gain-framed leaflet. The outcome measure was attendance at an NHS Health Check by November 2018 at active practices (in Lewisham, the outcome measure was attendance within 6 months of receiving the invitation, if that was sooner than the end of the trial). The study was powered to detect a 2% effect (which was considered to be the smallest meaningful effect size) at the 0.5% significance level between any two arms at 80% power with a baseline uptake of 38% (which was the percentage uptake in both Lewisham and NE Lincolnshire in 2017/8).

**Fig. 1:**
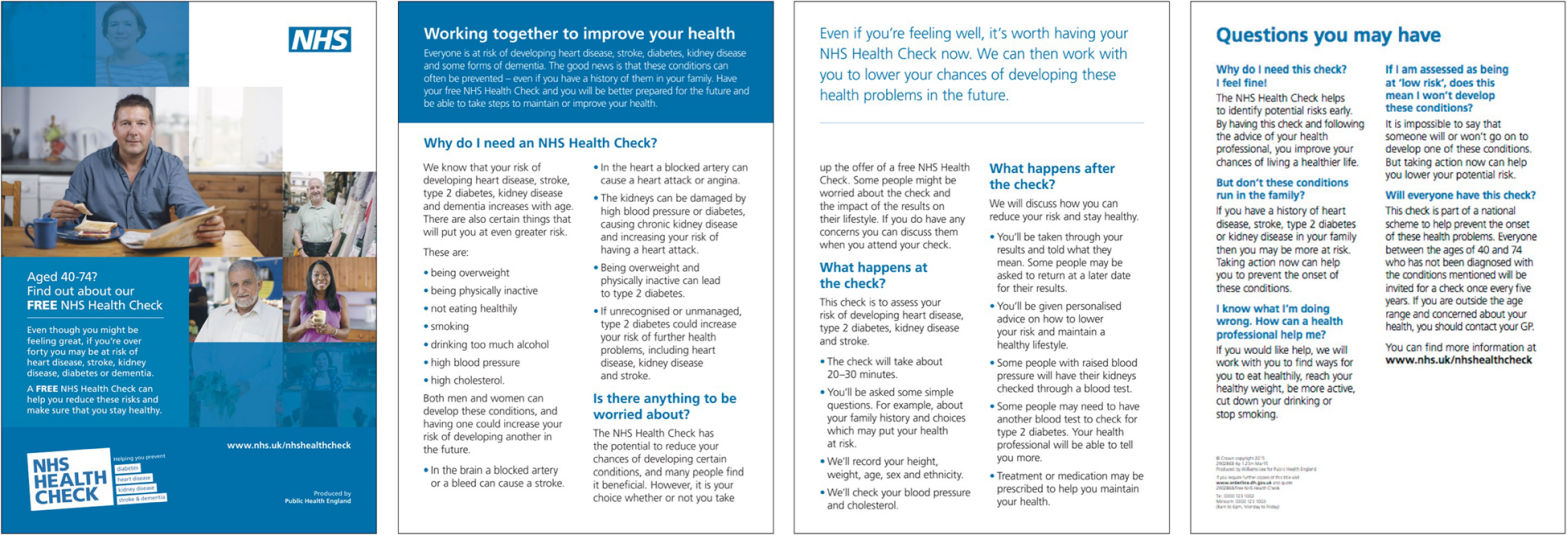
The control leaflet: the current national leaflet

The control leaflet was the current national leaflet, which is a 4-sided A5 leaflet with information on why the patient needs a health check, what will happen during and after the NHS Health Check, and ‘myth busting’ common questions people have about health checks. (See Fig. 1 for images or the Additional file 1 for a full-sized colour version of the leaflet.) The front page has stock photographs of people of different genders and races and says:

Aged 40–74? Find out about your **FREE** NHS Health Check.

Even though you might be feeling great, if you’re over forty you may be at risk of heart disease, stroke, kidney disease, diabetes or dementia.

A **FREE** NHS Health Check can help you reduce these risks and make sure that you stay healthy.

The second page starts with a paragraph on ‘Working together to improve your health’, then the second and third pages have a series of questions, with the answers in bullet points: ‘Why do I need an NHS Health Check?’, ‘Is there anything to be worried about?’, ‘What happens at the Check?’, and ‘What happens after the check?’. The fourth page is a list of ‘Questions you may have’, with one-paragraph answers.

**Fig. 2:**
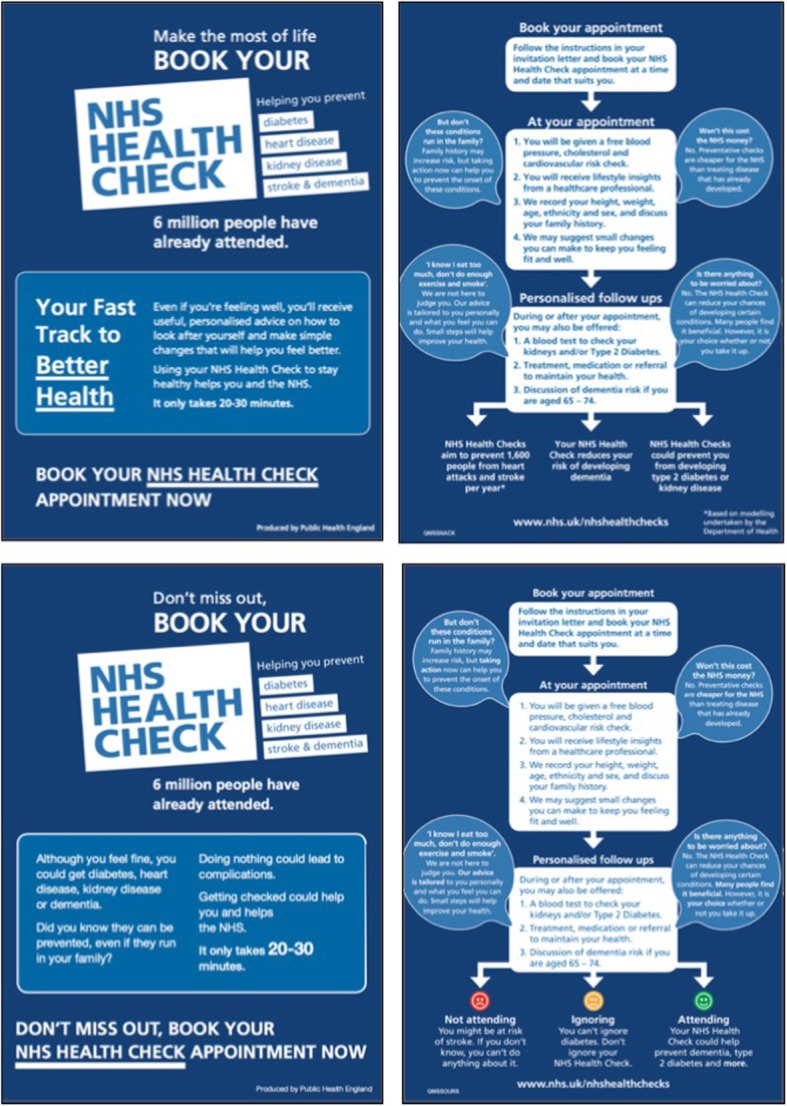
The intervention leaflets: Gain-framed leaflet at top, loss-framed leaflet at bottom

The intervention leaflets were designed by public health behavioural scientists, and scrutinised by PHE Behavioural Insights, PHE Marketing, and the PHE NHS Health Check Teams for: (1) required content to support informed decision making about the Health Check, (2) alignment with research on patient’s experiences of the NHS Health Check, and the barriers and facilitators of attendance, and (3) message framing as studied in the behavioural science literature. Both were 2-sided A5 flyers (See Fig. 2 for images or the Additional file 2 for full-sized colour versions of the leaflets.). The text on one side of the gain-framed leaflet stated:

Make the most of life. Book your NHS Health Check. 6 million people have already attended.

Your fast track to better health.

Even if you’re feeling well, you’ll receive useful personalised advice on how to look after yourself and make simple changes that will help you feel better. Using your NHS Health Check to stay healthy helps you and the NHS. **It only takes 20-30 minutes.**

**Book your NHS Health Check appointment now.**

The other side had a flowchart, showing what would happen during and after the health check process (instruction to book the appointment, what would happen at the appointment, and possible personalised follow ups), with ‘myth busting’ of common reasons that people do not go to health checks in speech bubbles. The flow chart ended by saying that the ‘NHS Health Checks aim to prevent 1,600 people from heart attacks and stroke per year’, ‘Your NHS Health Check reduces your risk of developing Dementia’, and ‘NHS Health Checks could prevent you from developing type 2 diabetes’.

The loss-framed leaflet had on one side:

Make the most of life. Book your NHS Health Check. 6 million people have already attended.

Although you feel fine, you could get diabetes, heart disease, kidney disease or dementia. Did you know they can be prevented, even if they run in your family? Doing nothing could lead to complications. Getting checked could help you and helps the NHS. **It only takes 20-30 minutes.**

**Don’t miss out, book your NHS Health Check appointment now**

The other side of the loss-framed leaflet was a flowchart like that on the gain-framed leaflet, except that instead of having the statistics at the end, it had traffic-light coloured faces with appropriate emotional expressions to reinforce the personalised consequences of the NHS Health Check: ‘Not attending: You might be at risk of stroke. If you don’t know, you can’t do anything about it’, ‘Ignoring: You can’t ignore diabetes. Don’t ignore your NHS Health Check’, and ‘Attending: Your NHS Health Check could help prevent dementia, type 2 diabetes and more.’ Colour copies of all three leaflets are available as Additional files 1 and 2.

### Ethical approval

Ethical approval was granted by the Proportionate Review Sub-Committee of the London-Hampstead Research Ethics Committee, REC reference 18/LO/0528, IRAS project ID 242350. The trial is registered at clinicaltrials.gov, identifier NCT03524131.

### Participants and eligibility

There were 11,831 participants: 8885 patients from 39 practices in Lewisham and 2946 patients from 17 practices in NE Lincolnshire, who received one of the leaflets with their NHS HC invitation letter from April–September 2018. Patients were eligible to take part in the trial if they were due to be invited for an NHS Health Check and met the NHS Health Check eligibility criteria: aged 40–74, do not have an existing cardiovascular-related medical condition, and have not had an NHS Health Check in the last 5 years.

### Procedure and randomisation

Invitations for the practices in Lewisham were sent by Quality Medical Solutions (QMS) and invitations for the practices in North East Lincolnshire were sent by the local authority administrator (although in both areas the letters were from the patient’s GP). Apart from using the new leaflets, other procedures were as normal in both localities.

In Lewisham, trial leaflets were sent out from March 2018, landing at the beginning of April, to August 2018, landing at the beginning of September (6 months). Data was collected until the end of November 2018, to take into account any time lag between getting the letter and making an appointment, as well as the period between making the appointment and the appointment itself. Reminders to participants were sent out 3 months after the initial invitation letter, if they had not yet completed an NHS Health Check. Patients whose mobile number was on record received a reminder text, patients without a mobile number received a second letter and leaflet. A patient who had an NHS Health Check within 6 months of receiving the invitation and before the end of the trial was recorded as attending. (A patient who attended later than 6 months after receiving their invitation would be recorded as an opportunistic check and would not be in the data set. So, for example, patients who received their invitation at the beginning of April would only be recorded as having attended if they had a Health Check before the beginning of October.)

In North East Lincolnshire, trial leaflets were sent in Q1, April–June, and Q2, July–September (6 months). Letters were sent out in the first 8 weeks of each quarter, then practices were given a list of their patients who had been invited, and they were encouraged to use the last month of the quarter to call any of those patients who had not booked in for a health check. Anonymised patient-level data on uptake was provided by QMS and North East Lincolnshire administration for the period between April and November for Lewisham and for Q1 and Q2 in North East Lincolnshire.

In each of Lewisham and North East Lincolnshire, patients were pseudo-randomised into three groups. Patients were assigned to one of the three trial groups based on the value of a pseudo-random number generated for each patient at the time that the patient was approved for an invitation. Both GPs and patients were blinded to intervention assignment and, indeed, to the fact that they were a part of a trial; the Research Ethics Committee agreed that patient consent was not required.

### Outcome measure

The outcome measure was attendance at the NHS Health Check, as recorded by individual practices. In addition, the GP practices provided anonymised data on age, sex, ethnicity, and previous health check invitation and attendance.

### Statistical analyses

Non-parametric tests (due to data not being normally distributed) were used to test for differences in age and sex across locations and leaflet conditions. A Chi-squared test of association was conducted to test for significant differences in uptake between leaflet conditions. After we saw the results, we decided post hoc to calculate the Bayes factor for the Chi-squared test, which was done using the Bayesian contingency test in JASP version 0.9.2 downloaded from: https://jasp-stats.org/.

A mixed effects logistic regression model was estimated, using SPSS version 23, in order to test whether the treatment leaflets increased uptake of the NHS Health Check in comparison to the control leaflet. The outcome variable was whether the participant attended the health check. The main independent variable was leaflet version; the national template (control leaflet) was used as the reference category. Age and sex were also entered into the regression, and GP practice was included as a random effect. Ethnicity was not modelled in the analysis because of the high proportion of missing data in North East Lincolnshire. A second mixed effects logistic regression model was estimated to test for effect of previous NHS Health Check attendance as well as two-way interaction effects between leaflet and age, sex, previous attendance.

## Results

### Descriptive statistics

**Fig. 3:**
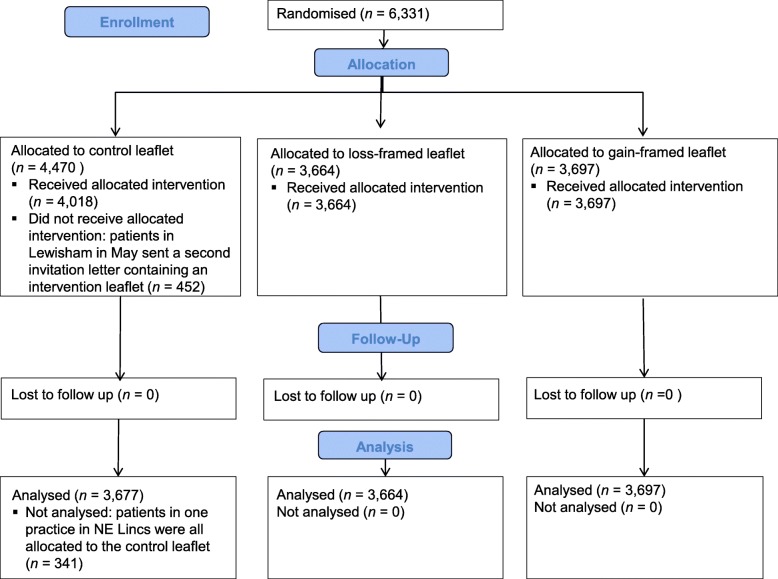
for the CONSORT flowchart

The final sample included 11,038 patients: 2605 patients from 17 GP practices in North East Lincolnshire and 8433 patients from 39 GP practices in Lewisham (one practice in Lewisham closed during the study so data from only 38 practices was received, but the patients in the closed practice would have been allocated to another practice and all practices in the local authority were a part of the trial, so it would still be recorded in the data if those patients attended an NHS Health Check in the local authority of Lewisham). In North East Lincolnshire, 881 patients received the control leaflet, 841 received the loss-framed leaflet, and 883 received the gain-framed leaflet. In Lewisham, 2796 patients received the control leaflet, 2823 received the loss-framed leaflet and 2814 received the gain-framed leaflet. The data from North East Lincolnshire contained an error where all 341 patients in one of the practices were allocated to the control condition, so the data from these patients was removed before analysis. In Lewisham, 452 patients in the control group for May were sent both the control leaflet and a second invitation with one of the intervention leaflets, so these patients were removed from the trial and adjustments were made to the trial groups in June, in order to have an even number of patients in each arm (control leaflet *n =* 3677, loss-framed *n =* 3664, gain-framed *n =* 3697). See Fig. 3 for the CONSORT flowchart.

The sample consisted of 5430 males (1596 in North East Lincolnshire and 3834 in Lewisham) and 5608 females (1009 in North East Lincolnshire and 4599 in Lewisham). Sex did not differ significantly between the three leaflet conditions, *Χ*^*2*^ (2, *N* = 11,038) *=* .43, *p* = .81, although it differed significantly between the two locations *Χ*^*2*^ (1, *N* = 11,038) *=*198.85, *p* < .001. Across the two locations age ranged from 39 to 74, with a mean of 50.42 (SD = 9.44) and median of 49. Age did not differ significantly between the three leaflet conditions, *Χ*^*2*^ (2, *N* = 11,038) *=* .52, *p* = .77. In North East Lincolnshire, age ranged from 40 to 74, with a mean of 57.81 (SD = 8.87) and median of 58. In Lewisham, age ranged from 39 to 74, with the mean of 48.13 (SD = 8.37) and median of 46. Age differed significantly between locations (*U* = 4,692,339.00, *Z* = − 44.73, *p* < .001). The demographics are summarized in Table 1.

**Table 1:** Demographics of the different trial arms, also broken down by trial area

	Control leaflet		Loss-framed leaflet		Gain-framed leaflet	
	NE Lincs.	Lewisham	NE Lincs.	Lewisham	NE Lincs.	Lewisham
Sex (male) n (% in each condition)	520 (59.0%)	1273 (45.5%)	517 (61.5%)	1290 (45.7%)	559 (63.3%)	1271 (45.2%)
	1793 (48.8%)		1807 (49.3%)		1830 (49.5%)	
Age at time of invitation (years) mean (s.d*.*)	58.0 (8.7)	48.1 (8.4)	57.5 (8.8)	48.2 (8.5)	57.9 (9.0)	48.0 (8.2)
	50.5 (9.4)		50.3 (9.4)		50.3 (9.4)	
Total patients	881	2796	841	2823	883	2814
	3677		3664		3697	

### Uptake of NHS Health check

**Fig. 4:**
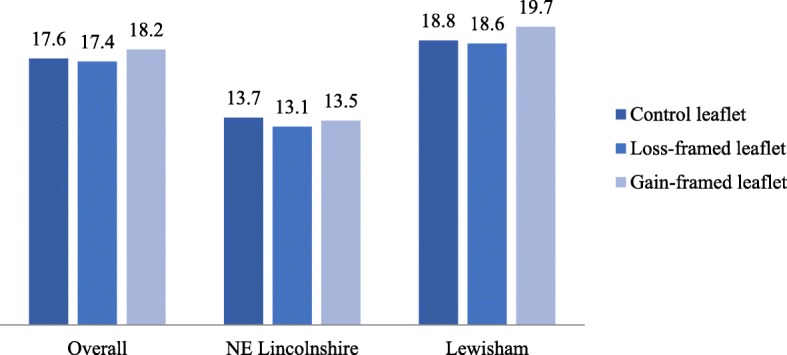
Percentage uptake of NHS Health Check across leaflet conditions

Overall, 1957 (17.7%) participants across the three arms of the trial attended an NHS Health Check. Of those who were sent the standard leaflet, 648 (17.6%) participants attended, 636 (17.4%) participants attended when sent the loss-framed leaflet and 673 (18.2%) participants attended when sent the gain-framed leaflet. See Table 2 and Fig. 4.

**Table 2:** Uptake of the NHS Health Check across trial arms, also broken down by trial area

	Control leaflet		Loss-framed leaflet		Gain-framed leaflet	
	NE Lincs.	Lewisham	NE Lincs.	Lewisham	NE Lincs.	Lewisham
*n* (number of patients whose data was analysed)	881	2796	841	2823	883	2814
	3677		3664		3697	
Uptake (number of patients who attended an NHS Health Check)	121	527	110	526	119	554
	648		636		673	
% Uptake	13.7%	18.8%	13.1%	18.6%	13.5%	19.7%
	17.6%		17.4%		18.2%

A Chi-square test indicated that NHS Health Check uptake was not significantly different across leaflet conditions, *Χ*^*2*^ (2, *N* = 11,038) *=* .95, *p* < .62. The Bayes factor for the null hypothesis over the alternative hypothesis (BF01) is 416, indicating that this result is 416 times more likely given the null hypothesis that the leaflets do not affect uptake than given the alternative hypothesis that they do. A Bayes Factor greater than 100 is generally considered ‘extreme’ evidence in favour of the null hypothesis [35–37].

A mixed effects logistic regression model with leaflet type, location, sex and age entered as fixed effects and GP practice entered as a random effect is significant overall *F*(5, 11,032) = 17.71, *p* < .001. However, leaflet type is not a significant predictor in the model *F*(2, 11,032) = .68, *p* = .51, indicating that neither intervention leaflet led to a statistically significant increase in uptake of the NHS Health Check as compared to the standard leaflet. Significant predictors in the model included: location *F*(1, 11,302) = 7.90, *p* = .005, *β* = −.47, with higher uptake in Lewisham (19.1%) than North East Lincolnshire (13.4%); sex *F*(1, 11,302) = 47.04, *p* < .001, *β* = −.36, with lower uptake in males (14.7%) compared to females (20.6%); and age *F*(1, 11,302) = 26.42, *p* < .001, *β* = .02, with higher uptake being associated with increasing age. See Table 3 for model coefficients. The random effect of GP practice was statistically significant (estimate = 0.24, *S.E.* = 0.06, *Z* = 3.83, *p* < .001).

**Table 3:** Fixed effects coefficients of mixed effects logistic regression

	Coefficient	Std. error	T	Sig.	95% confidence interval	
					Lower	Upper
Intercept	−2.46	.18	−14.01	<.001	−2.80	−2.12
Gain-framed leaflet	.05	.06	.80	.42	−.07	.17
Loss-framed leaflet	−.02	.06	−.32	.75	−.14	.10
NE Lincolnshire	−.47	.17	−2.81	.005	−.79	−.14
Male	−.36	.05	−6.86	<.001	−.46	−.26
Age	.02	.003	5.14	<.001	.01	.02

An additional model was estimated that included leaflet type, location, age, sex, previous NHS Health Check attendance, 2-way interactions between leaflet type and each of the other variables as fixed effects (with age entered as a standardized variable), and GP practice as a random effect. The overall model was significant *F*(14, 11,023) = 19.75, *p* < .001. The results remained aligned with those of the previous model, with leaflet type not being a significant predictor *F*(2, 11,023) = 0.01, *p* = .99, location being a significant predictor *F*(1, 11,023) = 10.90, *p* = .001, *β* = −.46 and sex being a significant predictor *F*(1, 11,023) = 36.31, *p* < .001, *β* = .32. However, age was no longer a significant predictor, *F*(1, 11,023) = 1.32, *p* = .96, *β* = −.003. Previous NHS Health Check attendance was a significant predictor *F*(1, 11,023) = 183.34, *p* < .001, *β* = −.76, with previous attendance being associated with a higher likelihood of uptake in the current trial. None of the 2-way interactions between leaflet and other variables were statistically significant in the model: leaflet type and location, *F*(2, 11,023) = .47, *p* = .62; leaflet type and sex, *F*(2, 11,023) = .00, *p* = 1.000; leaflet type and age, *F*(2, 11,023) = .50, *p* = .61; or leaflet type and previous attendance at NHS Health Check, *F*(2, 11,023) = 1.22, *p* = .30. See Table 4 for model coefficients. GP practice was not a significant random effect in this model (estimate = .23, *S.E*. = 0.06 *Z* = 3.85, *p* < .001).

**Table 4:** Fixed effects coefficients of mixed effects logistic regression including previous NHS Health Check attendance and 2-way interactions with leaflet type (age as a standardized variable)

	Coef.	Std. error	t	Sig.	95% confidence interval	
					Lower	Upper
Intercept	−0.81	.12	−6.50	<.001	−1.05	−.57
Gain-framed leaflet	0.12	.13	0.90	.37	−0.14	0.38
Loss-framed leaflet	0.15	.13	1.16	.25	−.11	.42
NE Lincolnshire	−.46	.19	−2.39	.017	−.84	−.08
Male	−.32	.09	−3.50	<.001	−.50	−.140
Age	−.003	.05	−.06	.96	−.11	.10
Previous NHS HC not completed	−.76	.11	−7.29	<.001	−.97	−.56
Gain-framed * NE Lincolnshire	−.17	.18	−.97	.33	−.52	.18
Loss-framed leaflet * NE Lincolnshire	−.07	.18	−.39	.70	−0.42	.28
Gain-framed leaflet * Male	−.001	.13	−.01	.99	−.25	.25
Loss-framed leaflet * Male	−.00	.13	−.002	.998	−.25	.25
Gain-framed leaflet * Age	.071	.072	.99	.32	−.07	.21
Loss-framed leaflet * Age	.005	.07	.63	.53	−.10	.19
Gain-framed leaflet * Previous HC not completed	−.05	.15	−.34	.73	−.33	.23
Loss-framed leaflet * Previous HC not completed	−.22	.15	−1.49	.14	−.50	.07

## Discussion

The aim of this trial was to compare the effectiveness of three leaflets on the uptake of NHS Health Checks by eligible patients in Lewisham and North East Lincolnshire: there were two new behaviourally-enhanced leaflets, one gain-framed and one loss-framed, and the current national leaflet (a standard practice control condition). The leaflets all accompanied the NHS Health Check invitation letter. There were no significant differences in uptake of the NHS Health Check between patients who received the gain-framed (18.2%), loss-framed (17.4%), or control (17.6%) leaflet with their NHS Health Check invitation letter, and the Bayes factor of 416 provides extreme evidence for the null hypothesis of no effect. Females, older patients, and patients in Lewisham were more likely to attend an NHS Health Check, though the effect of age disappeared when previous NHS Health Check attendance was entered into the model. There was significant variation between practices in the simpler model, but the variation became non-significant when previous NHS Health Check attendance was entered into the model. Overall, it can be concluded that changes to the leaflet were not successful at increasing uptake of NHS Health Checks; this result generalized across two very different areas, an inner London borough and an authority in the Yorkshire and Humber region in the East of England, which encompasses both towns and rural areas.

One explanation for the absence of an effect of the new behaviourally-enhanced leaflets compared to the national leaflet is that the behavioural insights used were not effective among the invited population. Alternatively, some of the insights applied might have been effective at increasing attendance, but some of them may have had the opposite effect from that intended, diminishing attendance and offsetting the successful changes. For instance, one technique that we used was simplification, but there is suggestive evidence that simplifying texts may only impact surface-level text processing and may even impede comprehension amongst readers with high background knowledge and low reading skills [38–40]. If the new leaflets decreased comprehension of the leaflet and therefore understanding of the NHS Health Check, then that might have deterred people from attending, offsetting any positive changes resulting from the other techniques that we applied. However, simplification has been effective in the context of the NHS Health Check, where simplified invitation letters were successful in increasing uptake [9–11]. Indeed, the revised invitation letters led to rather large increases in uptake, so another possibility is that there was a ceiling effect, with the revised letters achieving the maximum possible uptake from printed materials, leaving little scope for further increases in uptake by revising the leaflet (although we know from other work that SMSs and telephone calls can still increase uptake [10, 41]).

However, it is perhaps more likely that the trial found no effects because patient information leaflets are not effective at changing behaviour. To the extent that our new behaviourally-enhanced leaflets may have increased knowledge or improved informed consent, for instance if the flowchart makes the NHS Health Check process clearer, improved knowledge may not have led to improved uptake. A systematic review of patient information leaflets in screening suggests that leaflets may increase knowledge without affecting uptake [42] and modelling work suggests that knowledge was not a moderator of uptake in a diabetes screening trial [43]. This research is supported by a qualitative study, which found that patients do not think the information in screening leaflets would affect their choice whether to be screened, although it might affect their anxiety or satisfaction with the outcome [44]. There seems to be a difference in opinion about the function of patient information leaflets: practitioners think that increasing compliance is a prime function of leaflets, whereas patients view an informed decision not to comply as an acceptable outcome [45].

Where leaflets have an effect on actions, in addition to a letter, that may be because they increase self-efficacy for the action. One randomised controlled trial showed that an enhanced leaflet increased the rate of return of a screening test kit for bowel cancer [18], but the leaflet in that trial explained how to take the stool sample that patients needed to return for their faecal occult blood test (FOBT). Drawing on other research [46, 47], the authors suggest that the leaflet was effective because it enhanced participants’ perceived self-efficacy to complete the FOBT kit. In contrast, a questionnaire with a component that aimed to increase the self-efficacy for having an NHS Health Check was not effective at increasing uptake [48], plausibly because people do not have any doubts about their ability to attend an appointment. Further, although our new leaflets instructed people to book their NHS Health Check, we could not give specific instructions how to book, since booking procedures are different in different GP practices.

Another explanation for why the intervention leaflets were not effective is that people do not read the leaflets, but rather focus their attention on the letter. When the letters and leaflets were being developed, participants in focus groups usually read the letter first and used it to assess whether the leaflet would be relevant to them [14]. Potentially, patients had already made up their minds whether to attend after reading the letter, with those who had an interest in attending going on to read the leaflet, and with patients who felt they were unlikely to attend simply not reading the leaflet. It may also be the case that both the letter and the leaflet have a low impact. In one qualitative study of patients who did not attend their NHS Health Check, about a third of the participants did not even recall receiving the invitation [28].

In addition to the lack of effect of the behaviourally-enhanced leaflets compared to the control, this trial found no difference between a gain-framed and a loss-framed leaflet on the uptake of NHS Health Checks. Past research has found that the way in which health behaviour change appeals are framed can affect their effectiveness, with gain-frames being more effective at encouraging ‘prevention behaviours’, which prevent the onset of disease [33]. Assuming patients did read the leaflet but it had no effect, there are a variety of possible explanations for our failure to replicate this finding.

Patients may not view attending the NHS Health Check is as a prevention behaviour. Even though the NHS Health Check programme is preventive, by virtue of the assessments done, a proportion of attendees are diagnosed with health conditions and research shows that some patients wrongly expect to have a range of diagnostic tests [26, 27]. In that case, they might incorrectly view the attending the NHS Health Check as a ‘detection behaviour’, which detects the presence of a disease. Meta-analyses show that framing does not affect detection behaviours, such as attending screening appointments [49, 50]. Some research suggests that the effect of framing on health detection behaviours is moderated by individuals’ perception of their personal risk [49, 51, 52], and patients may not have perceived cardiovascular disease to be of high personal relevance.

Alternatively, the type of prevention behaviours promoted by the NHS Health Check may be different from those studied in the literature on the framing of prevention behaviour. Framing studies have focused on encouraging individuals to perform a healthy behaviour. However, the preventative aspect of the NHS Health Check involves both encouraging people to perform healthy behaviours (e.g., engaging in physical activity, eating healthily) and getting people to cease unhealthy behaviours (e.g., smoking cessation, reducing alcohol consumption, reducing consumption of unhealthy food, reducing sedentary behaviour). Whilst there is some evidence that gain-frames are more effective than loss-frames at encouraging people to perform healthy prevention behaviours, it is unclear whether gain-frames would also be more effective at encouraging people to stop performing an unhealthy behaviour [33, 34], or to attend a health check that aims to support people in giving up unhealthy behaviours.

The results of this trial are also consistent with evidence that there is only a very small effect of loss- versus gain-framing, which appears larger than it really is because of publication bias [49, 50, 53]. In one of the meta-analyses on detection behaviours, the authors note that the larger effects were found in smaller studies, which can be a sign of publication bias [50]. Some correlation between sample size and effect size has also been found in the prevention literature, although the authors concluded that this was not responsible for the significant effect of gain-frames on prevention [49]. Even if there is no publication bias, the effect sizes found in these meta-analyses are extremely small (− 0.04 [50], 0.03 [53], and 0.083 [49]), only detectable with sample sizes of thousands—which we did have, but many other studies in the literature do not.

Our findings that females and older patients were more likely to attend is consistent with other literature on the uptake of NHS Health Checks [9, 10, 54, 55]. Previous attendance also predicted uptake and, when it was added to the model, age was no longer a significant predictor. Age is associated with previous attendance because only over-40s are invited, so people who are older are more likely to have had a previous NHS Health Check invitation.

Uptake was higher in Lewisham than in North East Lincolnshire. The demographics of the two areas were different. North East Lincolnshire had a higher proportion of males, but it also had a higher mean age than Lewisham. However, our model showed that there was a difference in uptake between the two areas even when demographics were accounted for. There are many other reasons that could explain the differences in attendance: Lewisham is a borough in inner City London, and North East Lincolnshire is in the East of England and encompasses both small towns and rural areas. There are also differences in the NHS Health Check invitation process. Lewisham has a well-established NHS Heath Check invitation process, QMS has been sending the invitations for about 10 years, whereas in North East Lincolnshire the trial occurred in the first year of a new process, where the local authority sent out the invitations. The two locations had different reminder procedures. Text primers, as used in Lewisham, are effective at increasing uptake [10], so are phone calls, as used in North East Lincolnshire [56]. However, in North East Lincolnshire, making phone calls was left to individual GP practices and we do not know how many or which practices made the calls. Finally, the follow-up time in North East Lincolnshire was arguably shorter, half the patients were invited in the first month of Q2 and the final attendance was taken at the end of Q2 (a gap of 2 months), whereas in Lewisham 2 months was the smallest time period from invitation until the end of the trial and third of the patients had a 6-month follow-up period.

All these differences between the two areas and in their delivery of the NHS Health Check may explain why we found a difference in uptake between the areas. However, none of these differences will have affected the results of the trial because there were patients from Lewisham and NE Lincolnshire in both conditions and there was random allocation of patients to conditions. Therefore, all invitation methods and recall methods are expected to be equally represented in each trial condition. Further, there was no interaction effect between area and type of leaflet, so the most effective leaflet did not depend on the invitation procedures. In both areas, the follow-up period in the trial was shorter than the follow-up period for standard reporting, which may partly explain the decreased attendance compared to the previous year’s uptake of 38%. Seasonal variation in uptake may also have been a contributing factor, since data shows that take up dramatically increases in Q3 and Q4 [6].

Other studies also find significant variation between practices [41, 57–59]. Reasons for this may include different levels of engagement with the programme and different practice procedures for NHS Health Check appointment. In this study, both the variation among practices and the effect of age disappeared when previous NHS Health Check attendance was entered into the model. This is not surprising, since we would expect attendance at previous and current health checks to have common causes, and those causes would include both patient- and practice-level factors.

This study strongly suggests that future efforts at increasing uptake of the NHS Health Check should focus on avenues other than the leaflet. It also challenges the assumption that patient information leaflets can be used to influence behaviour, although we cannot tell if the lack of effect was due to the content of the leaflet, or because people did not read it, or because it continued to support informed decision making but this does not impact uptake. Patient information leaflets are still required for ethical reasons, as a part of a process of shared decision-making and informed consent, so future work might investigate comprehension of the NHS Health Check leaflet, how to improve it, and whether—as suggested by some research on healthcare resources [60, 61]—alternative formats including digital resources are as, if not more, effective at supporting attendance.

## Conclusions

We found no evidence for a meaningful effect of leaflet type on uptake, despite our trial being powered to detect a 2% difference. There was no significant difference between the behaviourally-enhanced leaflets and the current national leaflet, or between the gain-framed and the loss-framed leaflet. A Bayesian analysis showed that there is extreme evidence for the null hypothesis, of no differences in uptake between leaflets. This was the first trial to use behavioural insights in a patient information leaflet in order to influence attendance, as well as the first trial on the NHS Health Check leaflet; previous research on patient information leaflets has targeted knowledge, in the hope that an increase in informed consent would lead to an increase in uptake. However, our result is consistent with that body of research, in not finding an effect of leaflets on uptake. We found the lack of effect surprising, given that behaviourally informed letters have improved uptake of NHS Health Checks. It is possible that people do not pay attention to leaflets that are enclosed with letters or that their comprehension of the leaflets is poor or that the leaflet supports an informed decision not to attend. Further work could assess these hypotheses.

## Supplementary information

**Additional file 1:** NHS Health Check Current National Leaflet.

**Additional file 2:** NHS Health Check Intervention Leaflets.

## Data Availability

Data for this study is based on patient-level information collected by local authorities, as part of the NHS Health Check programme. Requests for the data need to be made via PHE’s Office for Data Release.

## Abbreviations

GP: General Practitioner
NHS: National Health Service
NHSHC: NHS Health Check
PHE: Public Health England
QMS: Quality Medical Solutions
