# Supplementary material for: Applying behavioural science to increase uptake of the NHS Health Check: a randomised controlled trial of gain- and loss-framed messaging in the national patient information leaflet
Source: BMC Public Health. 2019 Nov 14;19:1519. doi: 10.1186/s12889-019-7754-5 (PMC6854644; doi:10.1186/s12889-019-7754-5)
Supplement: Supplementary file 1 — Additional file 1: NHS Health Check Current National Leaflet. [file 12889_2019_7754_MOESM1_ESM.pdf]

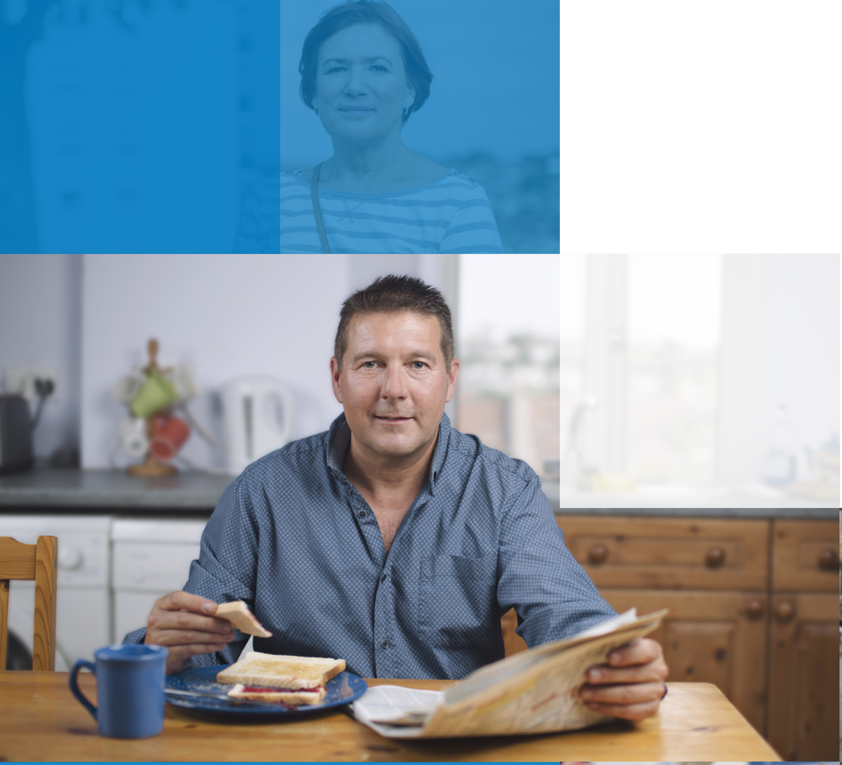

NHS

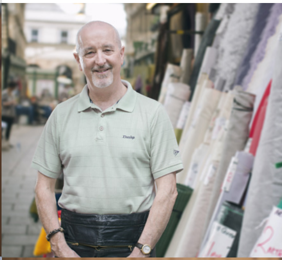

Aged 40-74?
Find out about our **FREE** NHS Health Check

Even though you might be feeling great, if you're over forty you may be at risk of heart disease, stroke, kidney disease, diabetes or dementia.

A **FREE** NHS Health Check can help you reduce these risks and make sure that you stay healthy.

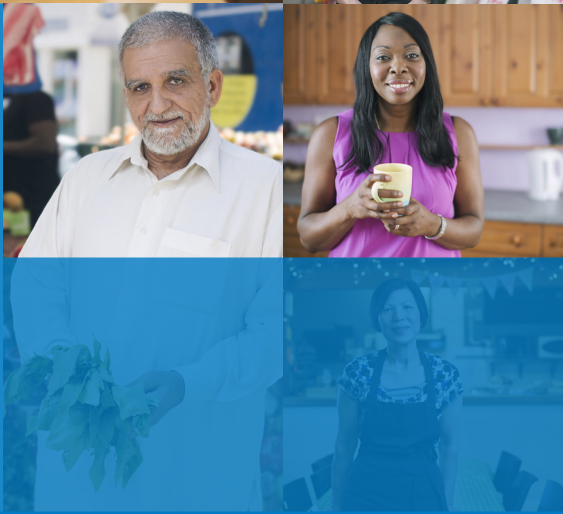

www.nhs.uk/nhshealthcheck

NHS HEALTH CHECK

Helping you prevent
- diabetes
- heart disease
- kidney disease
- stroke & dementia

Produced by
Public Health England

Working together to improve your health

Everyone is at risk of developing heart disease, stroke, diabetes, kidney disease and some forms of dementia. The good news is that these conditions can often be prevented – even if you have a history of them in your family. Have your free NHS Health Check and you will be better prepared for the future and be able to take steps to maintain or improve your health.

Why do I need an NHS Health Check?

We know that your risk of developing heart disease, stroke, type 2 diabetes, kidney disease and dementia increases with age. There are also certain things that will put you at even greater risk.

These are:

- being overweight
- being physically inactive
- not eating healthily
- smoking
- drinking too much alcohol
- high blood pressure
- high cholesterol.

Both men and women can develop these conditions, and having one could increase your risk of developing another in the future.

- In the brain a blocked artery or a bleed can cause a stroke.
- In the heart a blocked artery can cause a heart attack or angina.
- The kidneys can be damaged by high blood pressure or diabetes, causing chronic kidney disease and increasing your risk of having a heart attack.
- Being overweight and physically inactive can lead to type 2 diabetes.
- If unrecognised or unmanaged, type 2 diabetes could increase your risk of further health problems, including heart disease, kidney disease and stroke.

Is there anything to be worried about?

The NHS Health Check has the potential to reduce your chances of developing certain conditions, and many people find it beneficial. However, it is your choice whether or not you take

Even if you're feeling well, it's worth having your NHS Health Check now. We can then work with you to lower your chances of developing these health problems in the future.

---

up the offer of a free NHS Health Check. Some people might be worried about the check and the impact of the results on their lifestyle. If you do have any concerns you can discuss them when you attend your check.

What happens at the check?

This check is to assess your risk of developing heart disease, type 2 diabetes, kidney disease and stroke.

- The check will take about 20–30 minutes.
- You'll be asked some simple questions. For example, about your family history and choices which may put your health at risk.
- We'll record your height, weight, age, sex and ethnicity.
- We'll check your blood pressure and cholesterol.

What happens after the check?

We will discuss how you can reduce your risk and stay healthy.

- You'll be taken through your results and told what they mean. Some people may be asked to return at a later date for their results.
- You'll be given personalised advice on how to lower your risk and maintain a healthy lifestyle.
- Some people with raised blood pressure will have their kidneys checked through a blood test.
- Some people may need to have another blood test to check for type 2 diabetes. Your health professional will be able to tell you more.
- Treatment or medication may be prescribed to help you maintain your health.

Questions you may have

Why do I need this check? I feel fine!

The NHS Health Check helps to identify potential risks early. By having this check and following the advice of your health professional, you improve your chances of living a healthier life.

But don't these conditions run in the family?

If you have a history of heart disease, stroke, type 2 diabetes or kidney disease in your family then you may be more at risk. Taking action now can help you to prevent the onset of these conditions.

I know what I'm doing wrong. How can a health professional help me?

If you would like help, we will work with you to find ways for you to eat healthily, reach your healthy weight, be more active, cut down your drinking or stop smoking.

If I am assessed as being at 'low risk', does this mean I won't develop these conditions?

It is impossible to say that someone will or won't go on to develop one of these conditions. But taking action now can help you lower your potential risk.

Will everyone have this check?

This check is part of a national scheme to help prevent the onset of these health problems. Everyone between the ages of 40 and 74 who has not been diagnosed with the conditions mentioned will be invited for a check once every five years. If you are outside the age range and concerned about your health, you should contact your GP.

You can find more information at **www.nhs.uk/nhshealthcheck**

© Crown copyright 2015
2902868 4p 1.25m Mar15
Produced by Williams Lea for Public Health England

If you require further copies of this title visit **www.orderline.dh.gov.uk** and quote 2902868/Free NHS Health Check

Tel: 0300 123 1002
Minicom: 0300 123 1003
(8am to 6pm, Monday to Friday)
