# Supplementary material for: Applying behavioural science to increase uptake of the NHS Health Check: a randomised controlled trial of gain- and loss-framed messaging in the national patient information leaflet
Source: BMC Public Health. 2019 Nov 14;19:1519. doi: 10.1186/s12889-019-7754-5 (PMC6854644; doi:10.1186/s12889-019-7754-5)
Supplement: Supplementary file 2 — Additional file 2: NHS Health Check Intervention Leaflets. [file 12889_2019_7754_MOESM2_ESM.pdf]

Make the most of life

BOOK YOUR

NHS HEALTH CHECK

Helping you prevent

- diabetes
- heart disease
- kidney disease
- stroke & dementia

6 million people have already attended.

Your Fast Track to Better Health

Even if you're feeling well, you'll receive useful, personalised advice on how to look after yourself and make simple changes that will help you feel better.

Using your NHS Health Check to stay healthy helps you and the NHS.

It only takes 20-30 minutes.

BOOK YOUR NHS HEALTH CHECK APPOINTMENT NOW

Produced by Public Health England

Book your appointment

Follow the instructions in your invitation letter and book your NHS Health Check appointment at a time and date that suits you.

At your appointment

1. You will be given a free blood pressure, cholesterol and cardiovascular risk check.
2. You will receive lifestyle insights from a healthcare professional.
3. We record your height, weight, age, ethnicity and sex, and discuss your family history.
4. We may suggest small changes you can make to keep you feeling fit and well.

But don't these conditions run in the family?
Family history may increase risk, but taking action now can help you to prevent the onset of these conditions.

Won't this cost the NHS money?
No. Preventative checks are cheaper for the NHS than treating disease that has already developed.

Personalised follow ups

During or after your appointment, you may also be offered:

1. A blood test to check your kidneys and/or Type 2 Diabetes.
2. Treatment, medication or referral to maintain your health.
3. Discussion of dementia risk if you are aged 65 – 74.

'I know I eat too much, don't do enough exercise and smoke'.
We are not here to judge you. Our advice is tailored to you personally and what you feel you can do. Small steps will help improve your health.

Is there anything to be worried about?
No. The NHS Health Check can reduce your chances of developing certain conditions. Many people find it beneficial. However, it is your choice whether or not you take it up.

NHS Health Checks aim to prevent 1,600 people from heart attacks and stroke per year*

Your NHS Health Check reduces your risk of developing dementia

NHS Health Checks could prevent you from developing type 2 diabetes or kidney disease

www.nhs.uk/nhshealthchecks

*Based on modelling undertaken by the Department of Health

Don’t miss out,

BOOK YOUR

NHS HEALTH CHECK

Helping you prevent

- diabetes
- heart disease
- kidney disease
- stroke & dementia

6 million people have already attended.

Although you feel fine, you could get diabetes, heart disease, kidney disease or dementia.

Did you know they can be prevented, even if they run in your family?

Doing nothing could lead to complications.

Getting checked could help you and helps the NHS.

It only takes 20-30 minutes.

DON’T MISS OUT, BOOK YOUR NHS HEALTH CHECK APPOINTMENT NOW

Produced by Public Health England

Book your appointment

Follow the instructions in your invitation letter and book your NHS Health Check appointment at a time and date that suits you.

But don't these conditions run in the family? Family history may increase risk, but **taking action** now can help you to prevent the onset of these conditions.

At your appointment

1. You will be given a free blood pressure, cholesterol and cardiovascular risk check.
2. You will receive lifestyle insights from a healthcare professional.
3. We record your height, weight, age, ethnicity and sex, and discuss your family history.
4. We may suggest small changes you can make to keep you feeling fit and well.

Won't this cost the NHS money? No. Preventative checks are **cheaper for the NHS** than treating disease that has already developed.

'I know I eat too much, don't do enough exercise and smoke'. We are not here to judge you. **Our advice is tailored** to you personally and what you feel you can do. Small steps will help improve your health.

Personalised follow ups

During or after your appointment, you may also be offered:

1. A blood test to check your kidneys and/or Type 2 Diabetes.
2. Treatment, medication or referral to maintain your health.
3. Discussion of dementia risk if you are aged 65 – 74.

Is there anything to be worried about? No. The NHS Health Check can reduce your chances of developing certain conditions. **Many people find it beneficial.** However, it is **your choice** whether or not you take it up.

Not attending

You might be at risk of stroke. If you don't know, you can't do anything about it.

Ignoring

You can't ignore diabetes. Don't ignore your NHS Health Check.

Attending

Your NHS Health Check could help prevent dementia, type 2 diabetes and **more.**

www.nhs.uk/nhshealthchecks
